# Hydroxy Selenomethionine Alleviates Hepatic Lipid Metabolism Disorder of Pigs Induced by Dietary Oxidative Stress via Relieving the Endoplasmic Reticulum Stress

**DOI:** 10.3390/antiox11030552

**Published:** 2022-03-15

**Authors:** Jinzhong Jing, Shenggang Yin, Yan Liu, Yonggang Liu, Longqiong Wang, Jiayong Tang, Gang Jia, Guangmang Liu, Gang Tian, Xiaoling Chen, Jingyi Cai, Bo Kang, Hua Zhao

**Affiliations:** 1 Key Laboratory for Animal Disease-Resistance Nutrition of Ministry of Education, China Ministry of Agriculture and Rural Affairs of Sichuan Province, Animal Nutrition Institute, Sichuan Agricultural University, Chengdu 611130, China; jinzhong0214@163.com (J.J.); yin_shenggang@163.com (S.Y.); liuyan.2013@foxmail.com (Y.L.); 18380485189@163.com (L.W.); tangjiayong1005@163.com (J.T.); jiagang700510@163.com (G.J.); liugm@sicau.edu.cn (G.L.); 13555@sicau.edu.cn (G.T.); xlchen@sicau.edu.cn (X.C.); 11890@sicau.edu.cn (J.C.); 2 Adisseo Asia Pacific Pte. Ltd., Singapore 188778, Singapore; kevin.liu@adisseo.com; 3 College of Animal Science and Technology, Sichuan Agricultural University, Chengdu 611130, China; bokang@sicau.edu.cn

**Keywords:** OH-SeMet, dietary oxidative stress, endoplasmic reticulum stress, lipid metabolism disorder, selenotranscriptome, growing-finishing pigs

## Abstract

This study used 40 castrated male pigs to determine the protective effects of a new selenium molecule (hydroxy selenomethionine, OH-SeMet) on dietary oxidative stress (DOS) induced hepatic lipid metabolism disorder, and corresponding response of selenotranscriptome. The pigs were randomly grouped into 5 dietary treatments and fed a basal diet formulated with either normal corn and oils or oxidized diet in which the normal corn and oils were replaced by aged corn and oxidized oils, and supplemented with OH-SeMet at 0.0, 0.3, 0.6 and 0.9 mg Se/kg for a period of 16 weeks (*n* = 8). The results showed that DOS induced liver damage, increased serum alanine aminotransferase (ALT) and alkaline phosphatase (ALP) levels, decreased serum triacylglycerol (TG) level, suppressed antioxidant capacity in the liver, and changed lipid metabolism enzyme activity, thus causing lipid metabolism disorder in the liver. The DOS-induced lipid metabolism disorder was accompanied with endoplasmic reticulum (ER) stress, changes in lipid metabolism-related genes and selenotranscriptome in the liver. Dietary Se supplementation partially alleviated the negative impact of DOS on the lipid metabolism. These improvements were accompanied by increases in Se concentration, liver index, anti-oxidative capacity, selenotranscriptome especially 11 selenoprotein-encoding genes, and protein abundance of GPX1, GPX4 and SelS in the liver, as well as the decrease in SelF abundance. The Se supplementation also alleviated ER stress, restored liver lipid metabolism enzyme activity, increased the mRNA expression of lipid synthesis-related genes, and decreased the mRNA levels of lipidolysis-related genes. In conclusion, the dietary Se supplementation restored antioxidant capacity and mitigated ER stress induced by DOS, thus resisting hepatic lipid metabolism disorders that are associated with regulation of selenotranscriptome.

## 1. Introduction

The liver is the major metabolic organ in mammals, playing a crucial role in the metabolism of lipids. Hepatic disorder in lipid metabolism is a major risk factor in many diseases, such as hyperlipemia and fatty liver disease [1]. The current leading theories posit that one of the main causes of abnormal lipid metabolism is oxidative stress (OS) [2,3], although the regulation of OS on lipid metabolism is diverse under different physiological statuses. Most published studies on lipid metabolism suggest that OS promotes production of reactive oxygen species and suppresses antioxidant capacity, thus causing liver injury and lipid accumulation [4,5]. However, study also manifests that OS attenuates lipid synthesis and facilitates β-oxidation in hepatoma cells [6]. The endoplasmic reticulum (ER) represents a complex membranous network that plays an important role in the lipid synthesis and transportation [7,8], and ER homeostasis is essential for lipid metabolism. OS compromises ER homeostasis, leading to a situation called ER stress [9]. It is very well established that OS usually synergistically affects lipid metabolism alongside ER stress [2,10].

Among many biomarkers for evaluating ER stress, unfolded protein response (UPR) appears to be representative [11]. The major outcomes of UPR activation are to alleviate ER stress and enhance the folding capacity of the ER [12]. The UPR is mediated by three ER transmembrane sensors: inositol-requiring protein 1 (IRE1), protein kinase-like endoplasmic reticulum kinase (PERK) and activating transcription factor 6 (ATF6) [13]. The unfolded proteins promote the phosphorylation of IRE1, which in turn lead to X-box-binding protein 1s (XBP1s) activation, thus promoting the occurrence of UPR [14,15]. The structure and function of PERK are similar to IRE1, and the activated PERK phosphorylates the α-subunit of the eukaryotic translation initiation factor 2 alpha (eIF2α), which attenuates ER load [16,17]. ER stress promotes the migration of ATF6 from the ER to Golgi and gets cleaved by serine proteases into active ATF6f [18], which can enter the nucleus and activate UPR [19]. In any case, abundant ER luminal unfolded proteins activate the UPR and cause downstream reactions, thus accelerating the resolution of ER stress [20]. It is thus reasonable to expect that mitigation of OS and ER stress may be an effective strategy to maintain lipid metabolic homeostasis.

Selenium (Se) is the key component of the body’s antioxidant system, which plays a crucial role in scavenging free radicals and maintaining antioxidant capacity [21]. Also, several previous studies posit that dietary Se deficiency or excessive Se (3 mg Se/kg) accelerates lipid synthesis in the mammalian liver, while the normal level of Se (0.3–1.0 mg/kg) exhibits no impact on lipid metabolism [22,23,24]. Current leading theories assume the main biological functions of Se are predominantly mediated by selenoproteins [25]. At present, 25 selenoproteins have been found in mammals and 7 (DIO2, SelF, SelK, SelM, SelN, SelS and SelT) of them are located in the ER [26]. There are few studies on the regulation of selenoproteins in lipid metabolism. A previous study reports that SelS attenuates ER stress during the early stage of adipogenesis [27], and SelK deficiency induces ER stress in neurons [28]. In view of the special relation between selenoproteins and ER function, it is logical to expect that some selenoproteins may alleviate the hepatic disorder of lipid metabolism induced by OS through relieving the ER stress. Generally, the expression of selenoproteins in animals is effectively regulated by the dietary Se level [29], and the bioavailability of organic Se is more efficient than its inorganic counterpart [21]. Hydroxy selenomethionine (OH-SeMet) is a new molecule of organic Se and is the precursor of selenocysteine [30]. Therefore, this study focuses on the regulation of OS on ER stress and hepatic lipid metabolism, and the protective role of OH-SeMet on the lipid metabolism homeostasis through regulating the selenotranscriptome.

## 2. Materials and Methods

### 2.1. Animal, Diet and Experimental Design

The animal trial was approved by the Animal Care and Use Committee of the Sichuan Agricultural University (Ethics Approval Code: SCAUAC201904-4).

A total of 40 castrated male pigs (Duroc × Landrace × Yorkshire, average live weight 25.0 ± 3.0 kg) were randomly grouped into 5 dietary treatments with 8 replicates of one pig per replicate. Before entering the designated dietary treatment, the pigs were fed on basal diet (using normal corn and normal oils, without additional Se supplementation) for 7 days to balance the body Se level. During the experiment, the animals were fed on basal diet (control group, CON) or oxidized diet (the normal corn and oils in basal diet were replaced by aged corn stored over 4 years and oxidized oils), supplied with 0.0 (dietary oxidative stress group, DOS), 0.3 (DOS + 0.3 Se), 0.6 (DOS + 0.6 Se) and 0.9 mg Se/kg (DOS + 0.9 Se) in the form of OH-SeMet for 16 weeks. The basal diet in the current study was formulated in accordance with NRC 2012 (Supplementary Table S1); the determined dietary Se concentration was shown in Supplementary Figure S1. The pigs were penned in fattening circle house with free access to diet and water, and the house temperature was maintained at 25 ± 2 °C and 20 ± 2 °C for 25–50 kg and >50 kg, respectively. The preparation method of oxidized oil was reported in the previous study [31]. The oxidation characteristics of the diets are shown in Supplementary Table S2.

### 2.2. Sample Collection and Preparation

After 16 weeks, blood samples were collected in sterile vacutainer tubes from the jugular vein and maintained at room temperature for 1 h, centrifuged (3000× *g*) for 10 min, and the serum samples were pipetted into sterile centrifuge tubes. All serum samples were stored at −20 °C prior to further analyses. Six pigs per treatment were selected and fasted for 12 h, and then weighed and sacrificed by electrical shock and exsanguinated. The livers of these pigs were weighed, and samples were collected and snap-frozen in liquid nitrogen, then stored at −80 °C for biochemical and molecular analyses.

### 2.3. Liver Phenotypic Characteristics and Liver Se Concentration

After the pigs were slaughtered, all livers were completely removed and weighed after removing the gallbladder, which were recorded as liver weight. The liver indexes were estimated: liver index = liver weight/pig’s live weight × 100% [32]. The concentration of Se in the liver was measured by using hybrid generation-atomic fluorescence spectrometer (AFS-230E, Beijing Haiguang instrument, China) against a standard Se reference [GB 5009.93-2010, National Research Centre for Certified Reference Materials, Beijing, China].

### 2.4. Serum Biochemical Analyses

Serum alanine aminotransferase (ALT), aspartate aminotransferase (AST), alkaline phosphatase (ALP), total protein (TP), albumin (ALB), globulin (GLB), triacylglycerol (TG), total cholesterol (TC), low density lipoprotein cholesterin (LDL-C), high density lipoprotein cholesterin (HDL-C) were measured using the automatic biochemistry analyzer (3100, HITACHI, Tokyo, Japan).

### 2.5. Antioxidant and Lipid Metabolic-Related Enzyme Analyses

Glutathione peroxidase (GSH-Px), superoxide dismutase (SOD), total antioxidant capability (T-AOC) and malondialdehyde (MDA) in the liver were measured by using corresponding assay kits (no. A005, A001-1-1, A015-1, A003-1, Jiancheng Bioengineering, Nanjing, China). The activity of acetyl-coa carboxylase (ACC), fatty acid synthase (FAS) and adipose triglyceride lipase (ATGL) in the liver were measured by using the commercial enzyme-linked immunosorbent assay (ELISA) kits (no. 15856, 17341, 14811, Meimian, China). The activity of hepatic lipase (HL) in the liver was determined by using commercial assay kit (no. A067-1-2, Jiancheng Bioengineering, Nanjing, China). The concentration of proteins was determined with the bicinchoninic acid (BCA) method by using a commercial BCA protein assay kit (no. A045-3, Jiancheng Bioengineering, Nanjing, China). The optical density (OD) values were obtained by the enzyme-labeling measuring instrument (Model 680, Bio-Rad, Hercules, CA, USA); all measurements were performed in triplicate.

### 2.6. Q-PCR Analyses of mRNA Abundance

The total RNA of liver was extracted by using RNAiso Plus (no. 9109, Takara, Dalian, China) and cDNA was synthesized with the PrimeScript RT reagent kit (no. RR047A, Takara, Dalian, China). The qPCR was performed in a final volume of 10 μL using SYBR Premix Ex TaqTM II kit (no. RR820A, Takara, Dalian, China) on the QuantStudio 6 Flex system (Applied Biosystems, Waltham, CA, USA). All samples of the target genes in a given tissue were run on the same 384-well plate (Applied Biosystems, Waltham, CA, USA). The primers for 17 lipid metabolic-related genes, 8 ER stress markers, 25 selenoprotein-encoding genes, and 2 reference genes (β-Actin and Gapdh) were designed with Primer Express 3.0 (Applied Biosystems, Waltham, CA, USA), and listed in Supplementary Table S3. The relative mRNA expression was normalized to the expression of β-Actin and calculated by using the 2^−ΔΔCt^ method as previously described [33].

### 2.7. Western Blot Analyses

The liver samples were homogenized with the cell disruption buffer (RIPA lysis Buffer, Beyotime, Shanghai, China). Then, the total protein concentration was measured using the BCA kit. The subsequent Western blot process was performed as previously described [32]. The primary antibodies were used at the following dilutions: eIF2α (1:500; 340347; Zen BioScience, Chengdu, China), P-eIF2α (1:500; 310073; Zen BioScience, Chengdu, China), XBP1 (1:1000; R27438; Zen BioScience, Chengdu, China), GRP78 (1:1000; 200310-4F11; Zen BioScience, Chengdu, China), GPX1 (1:1000; 616958; Zen BioScience, Chengdu, China), GPX4 (1:2000; 513309, Zen BioScience, Chengdu, China), DIO2 (1:500; 161625; Zen BioScience, Chengdu, China), SelF (1:1000; 385690; Zen BioScience, Chengdu, China), SelS (1:1000, 15591-1-AP, ProteinTech Group, Chicago, IL, USA) and β-ACTIN (1:5000; MAB1501; Millipore, Darmstadt, Germany).

### 2.8. Statistical Analysis

This study followed the complete random design (CRD) and applied the one-way structure treatment design. Six samples in a given tissue of each dietary treatment were tested to collect the data, except that liver Se concentration used 4 samples to collect the data. All values are presented as means ± SD. Statistical analyses were performed with the SPSS 27.0 (SPSS Inc., Chicago, IL, USA), values were analyzed using one-way ANOVA, followed by Tukey’s multiple range tests, and *p* values of less than 0.05 were considered statistically significant. Principal component analysis of selenotranscriptome in the liver and correlation analysis of the relationship between selenoprotein-encoding genes and lipid metabolism markers were accomplished by SPSS 27.0 (SPSS, Inc., Chicago, IL, USA).

## 3. Results

### 3.1. Liver Weight, Index and Se Concentration

**Figure 1:**
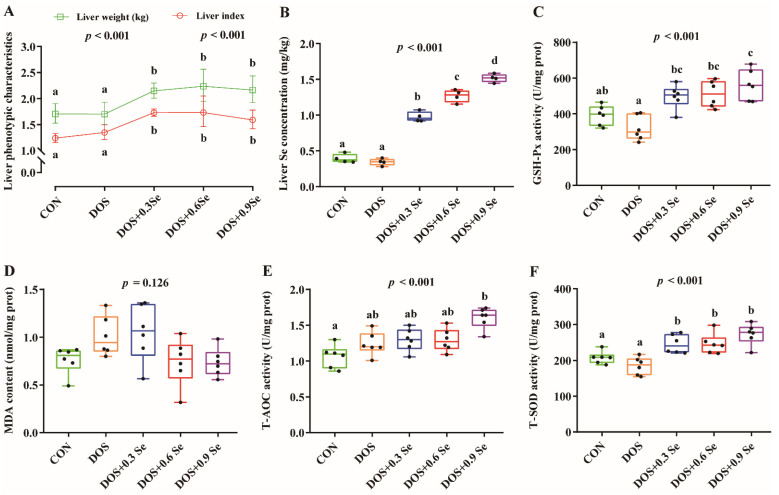
Effects of dietary oxidative stress and OH-SeMet supplementation on liver phenotypic characteristics, Se concentration and antioxidant capacity of growing-finishing pigs. (**A**) Liver phenotypic characteristics; (**B**) Liver Se concentration; (**C**) GSH-Px activity; (**D**) MDA content; (**E**) T-AOC activity; (**F**) T-SOD activity. Results were expressed as mean ± SD (*n* = 6 or 4), different letters indicate ANOVA *p* value less than 0.05 which represent there is a significant difference between groups.

As shown in Figure 1, DOS exhibited no effects (*p* > 0.05) on the absolute liver weight and liver index (Figure 1A). Relative to the DOS group, pigs received three levels of Se showed a higher (*p* < 0.05) liver weight and liver index. DOS alone showed no effect (*p* > 0.05) on liver Se concentration, while Se supplementation increased (*p* < 0.05) liver Se concentration in a dose dependent pattern (Figure 1B).

### 3.2. Hepatic Antioxidant Variables

To evaluate whether DOS induces oxidative damage in liver and the protective effects of Se, we further investigated four antioxidant attributes. DOS alone decreased the activity of GSH-Px to a certain extent (Figure 1C) and increased (*p* < 0.05) the activity of T-AOC (Figure 1E). Although there was no statistical difference, DOS tended to increase (*p* > 0.05) MDA content (Figure 1D). The Se supplementation improved the hepatic antioxidant capacity, as reflected by increased GSH-Px activity (*p* < 0.05) in a dose dependent pattern (Figure 1C), and increased activity of T-AOC and T-SOD (*p* < 0.05) (Figure 1E,F).

### 3.3. Serum Biochemical Indicators

The results of serum biochemical indicators are presented in Table 1. The *p* values of one-way ANOVA showed that DOS and OH-SeMet supplementation had significant influence on serum ALT, ALP and TG levels. The pigs faced with DOS showed higher serum ALT, ALP levels, and lower serum TG level. Se supplementation moderately ameliorated the negative effect of DOS on serum ALT, ALP and TG levels, especially Se 0.9 mg/kg decreased (*p* < 0.05) serum ALT level, Se 0.3 mg/kg decreased (*p* < 0.05) serum ALP level, and Se 0.6 and 0.9 mg/kg increased (*p* < 0.05) serum TG level. Besides, DOS alone showed a tendency to increase the concentration of serum AST, and Se supplementation restored the AST level in serum.

### 3.4. Lipid Metabolic-Related Enzyme Activity and Genes Expression in the Liver

**Figure 2:**
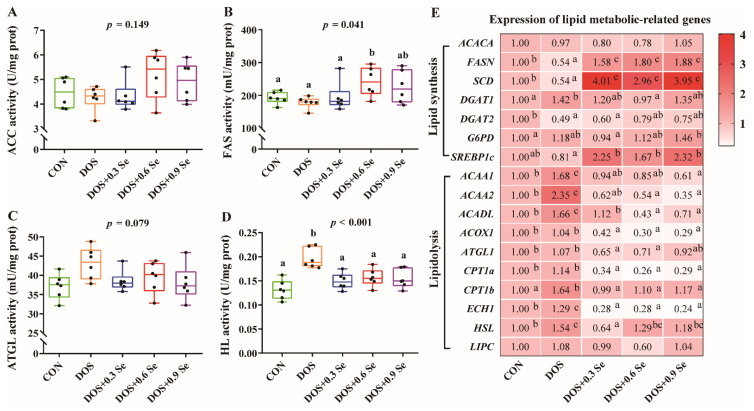
Effects of dietary oxidative stress and OH-SeMet supplementation on lipid metabolism related enzyme activity and expression of lipid metabolic-related genes in the liver of growing-finishing pigs. (**A**) Acetyl-coa carboxylase (ACC) activity; (**B**) Fatty acid synthase (FAS) activity; (**C**) Adipose triglyceride lipase (ATGL) activity; (**D**) Hepatic lipase (HL) activity; (**E**) Expression of lipid metabolic-related genes. Results were expressed as mean ± SD (*n* = 6), different letters indicate ANOVA *p* value less than 0.05 which represent there is a significant difference between groups.

We investigated lipid metabolic-related enzyme activity and genes expression in the liver (Figure 2). DOS increased (*p* < 0.05) the activity of HL (Figure 2D) and enhanced the activity of ATGL (Figure 2C) to a certain degree, while it exhibited limited impact (*p* > 0.05) on the activity of ACC and FAS (Figure 2A,B). Three levels of Se restored (*p* < 0.05) the activity of HL to normal levels (Figure 2D), 0.6 mg/kg Se increased (*p* < 0.05) the FAS activity (Figure 2B), and additional Se supplementation showed a tendency to reduce (*p* = 0.079) the ATGL activity (Figure 2C).

As shown in Figure 2E, for lipid synthesis-related genes, DOS down-regulated (*p* < 0.05) the mRNA abundance of fatty acid synthetase (*FASN*), stearoyl-CoA desaturase (*SCD*), diacylglyceryl transferase 2 (*DGAT2*), up-regulated (*p* < 0.05) the mRNA expression of diacylglyceryl transferase 1 (*DGAT1*) and exhibited no effect (*p* > 0.05) on the expression of *ACACA* and glucose-6-phosphate (*G6PD*). For lipidolysis-related genes, DOS up-regulated (*p* < 0.05) the mRNA expression of acetyl-coa acyltransferase 1 (*ACAA1*), acetyl-coa acyltransferase 2 (*ACAA2*), acyl-CoA long-chain dehydrogenase (*ACADL*), carnitine palmitoyltransferase 1b (*CPT1b*), enoyl-CoA hydratase 1 (*ECH1*) and hormone sensitive lipase (*HSL*), while it exhibited no impact (*p* > 0.05) on the expression of acetyl-coa oxidase 1 (*ACOX1*), adipose triglyceride lipase (*ATGL1*), carnitine palmitoyltransferase 1a (*CPT1a*) and hepatic lipase (*LIPC*). Dietary Se supplementation affected the expression of lipid metabolic-related genes. For lipid synthesis-related genes, three levels of Se increased (*p* < 0.05) the mRNA expression of *FASN*, *SCD* and sterol regulatory element binding protein 1c (*SREBP1c*), while exhibited limited impact on the expression of *ACACA*, *DGAT1* and *DGAT2*. For lipidolysis-related genes, three levels of Se down-regulated (*p* < 0.05) the mRNA abundance of *ACAA1*, *ACAA2*, *ACADL*, *ACOX1*, *ATGL1*, *CPT1a*, *CPT1b*, *ECH1* and *HSL*, with no effects on the expression of *LIPC*.

### 3.5. ER Stress Biomarkers in the Liver

**Figure 3:**
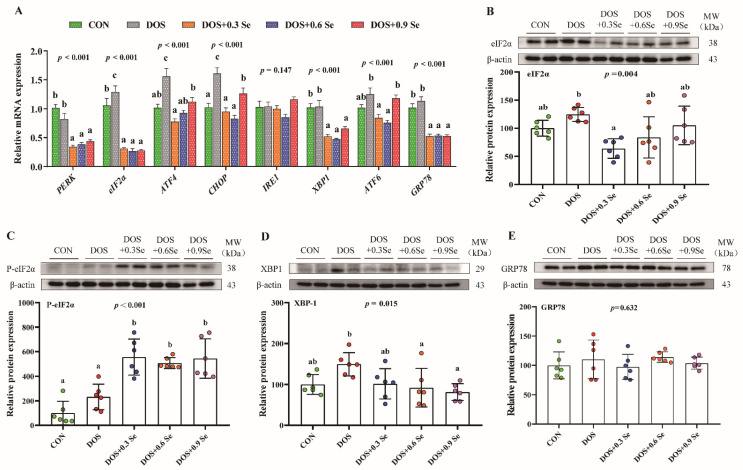
Effects of dietary oxidative stress and OH-SeMet supplementation on the ER stress biomarkers in the liver of growing-finishing pigs. (**A**) The mRNA abundance of *PERK*, *eIF2α*, *ATF4*, *CHOP*, *IRE1*, *XBP1*, *ATF6* and *GRP78*; (**B**) Relative protein abundance of eIF2α; (**C**) Relative protein abundance of P-eIF2α; (**D**) Relative protein abundance of XBP1; (**E**) Relative protein abundance of GRP78. Results were expressed as mean ± SD (*n* = 6), different letters indicate ANOVA *p* value less than 0.05 which represent there is a significant difference between groups.

The ER stress markers of un-folded protein response in liver were determined (Figure 3). Compared with the control group, DOS up-regulated (*p* < 0.05) the mRNA expression of *eIF2α*, *ATF4* and *CHOP*, and had a tendency (0.05 < *p* < 0.1) to increase the abundance of *ATF6*. The three levels of Se supplementation reduced (*p* < 0.05) the mRNA expression of *PERK*, *eIF2α*, *ATF4*, *CHOP*, *XBP1* and *GRP78*. Se 0.3 and 0.6 mg/kg reduced the levels of *ATF6*. DOS and Se showed no effect on the mRNA expression of *IRE1* (Figure 3A).

We further examined the ER stress markers at the protein level. The results showed that DOS had a tendency (0.05 < *p* < 0.1) to increase the protein level of eIF2α (Figure 3B) and XBP1 (Figure 3D). Dietary Se supplementation exhibited protective effects. Three levels of Se recovered the abundance of eIF2α, especially Se 0.3 mg/kg group showed the lowest eIF2α level (Figure 3B). Se supplementation also reduced (*p* < 0.05) the expression of XBP1 (Figure 3D) and increased (*p* < 0.05) the protein level of P-eIF2α (Figure 3C). On the other hand, DOS and Se exhibited no impact on the protein abundance of GRP78 (Figure 3E).

### 3.6. The Expression of Selenotranscriptome in the Liver

**Figure 4:**
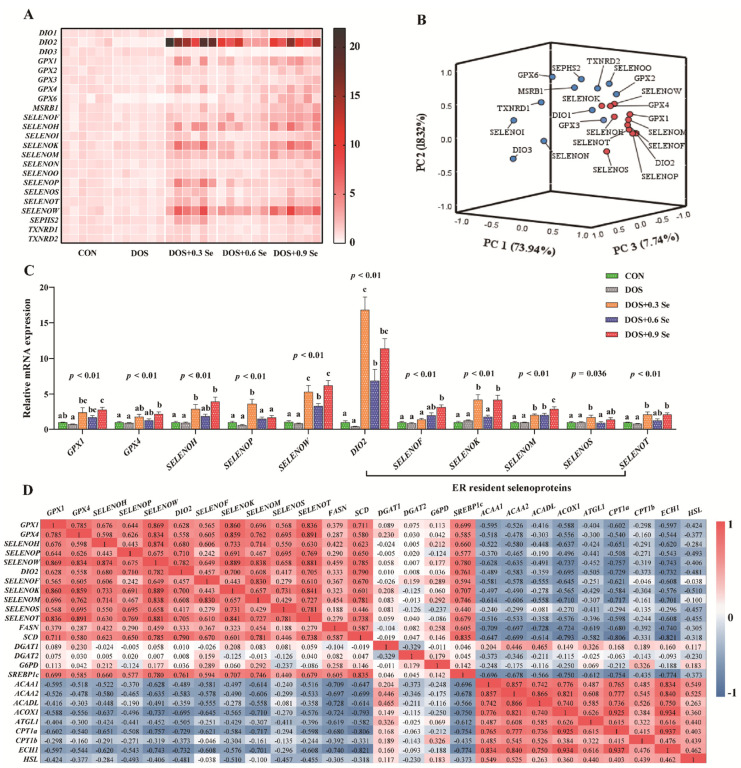
Effects of dietary oxidative stress and OH-SeMet supplementation on the expression of selenotranscriptome in the liver of growing-finishing pigs. (**A**) Heatmap of mRNA abundance of 23 selenoproteins; (**B**) Principal component analysis of the selenotranscriptome; (**C**) The mRNA abundance of *GPX1*, *GPX4*, *SELENOH*, *SELENOP*, *SELENOW*, *DIO2*, *SELENOF*, *SELENOK*, *SELENOM*, *SELENOS* and *SELENOT*; (**D**) The correlations analysis between selenotranscriptome and lipid metabolic-related genes. The results were expressed as mean ± SD (*n* = 6), different letters indicate ANOVA *p* value less than 0.05 which represent there is a significant difference between groups.

Se exerts its most-known biological functions mainly through selenoproteins. So, we investigated the expression of selenotranscriptome in the liver. *SELENOV* and *TXNRD3* were in poor quality or low expression and were not reported herein. As shown in Supplementary Figure S2 and Figure 4A, DOS exhibited limited impact on the mRNA expression of selenotranscriptome, except down-regulated (*p* < 0.05) the expression of *SELENON*. Dietary OH-SeMet supplementation, especially Se 0.3 and 0.9 mg/kg, generally up-regulated (*p* < 0.05) the mRNA abundance of 22 selenoprotein-encoding genes (*DIO1*, *DIO2*, *GPX1*, *GPX2*, *GPX3*, *GPX4*, *GPX6*, *MSRB1*, *SELENOF*, *SELENOH*, *SELENOI*, *SELENOK*, *SELENOM*, *SELENON*, *SELENOO*, *SELENOP*, *SELENOS*, *SELENOT*, *SELENOW*, *SEPHS2*, *TXNRD1* and *TXNRD2*), while it reduced (*p* < 0.05) the expression of *DIO3*.

### 3.7. The mRNA Expression of Major Selenoprotein-Encoding Genes

To distinguish the key factors affected by DOS and OH-SeMet, a principal component analysis was performed by a mathematical method of dimensionality reduction. As shown in Figure 4B, three comprehensive variables were chosen to reflect the original variable information, the three comprehensive variables were 73.94%, 18.32% and 7.74%, respectively. The results showed that 11 of 23 selenoprotein-encoding genes were observed at relatively distant positions in three-dimensional space. *GPX1*, *GPX4*, *SELENOH*, *SELENOP*, *SELENOW*, *DIO2*, *SELENOF*, *SELENOK*, *SELENOM*, *SELENOS* and *SELENOT* were the major selenoprotein-encoding genes affected by dietary OS and OH-SeMet levels, and in which 6 of 11 were ER resident selenoproteins. The mRNA abundance of those 11 selenoprotein-encoding genes were up-regulated (*p* < 0.05) in Se supplementation groups compared with the DOS group, except Se 0.6 and 0.9 mg/kg restored the mRNA expression of *SELENOP* (Figure 4C).

### 3.8. The Correlation Analysis between Selenotranscriptome and Lipid Metabolic-Related Genes

The correlation analysis was performed to explore the relationship between the major selenoprotein-encoding genes and lipid metabolism markers (details are provided in Supplementary Table S4). As shown in Figure 4D, significant positive correlations were found between the 11 selenoprotein-encoding genes and *SCD*/*SREBP1c* (Pearson correlation coefficient > 0.44; *p* < 0.05). Five selenoprotein-encoding genes (*GPX1*, *SELENOH*, *SELENOW*, *SELENOF* and *SELENOM*) were positively correlated with *FASN* (Pearson correlation coefficient > 0.367; *p* < 0.05). Besides, the major selenoprotein-encoding genes exhibited a negative correlation with *ACAA1*, *ACAA2*, *ACOX1*, *CPT1a* and *ECH1* (Pearson correlation coefficient < −0.370; *p* < 0.05), except *SELENOM*. Eight selenoprotein-encoding genes (*GPX1*, *SELENOH*, *SELENOP*, *SELENOW*, *DIO2*, *SELENOK*, *SELENOS* and *SELENOT*) were negatively correlated with *ATGL1* and *HSL* (Pearson correlation coefficient < −0.377; *p* < 0.05). Five selenoprotein-encoding genes (*GPX1*, *SELENOH*, *SELENOW*, *SELENOF* and *SELENOM*) were negatively correlated with *ACADL* (Pearson correlation coefficient < −0.416; *p* < 0.05).

### 3.9. The Protein Abundance of Selenoproteins

**Figure 5:**
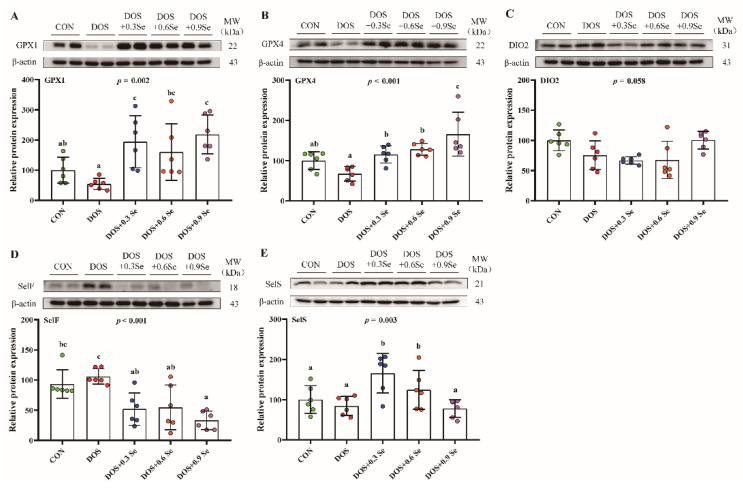
Effects of dietary oxidative stress and OH-SeMet supplementation on the expression of selenoproteins in liver of growing-finishing pigs. (**A**) Relative protein abundance of GPX1; (**B**) Relative protein abundance of GPX4; (**C**) Relative protein abundance of DIO2; (**D**) Relative protein abundance of SelF; (**E**) Relative protein abundance of SelS. The results were expressed as mean ± SD (*n* = 6), different letters indicate ANOVA *p* value less than 0.05 which represent there is a significant difference between groups.

The effects of DOS and OH-SeMet supplementation on protein abundance of GPX1, GPX4, DIO2, SelF and SelS were determined (Figure 5). Compared with the CON group, DOS tended to decrease (0.05 < *p* < 0.1) the protein level of GPX1 and GPX4, and to increase (0.05 < *p* < 0.1) the protein level of SelF. Dietary Se supplementation increased (*p* < 0.05) the protein abundance of GPX1 and GPX4, Se 0.3 and 0.6 mg/kg increased (*p* < 0.05) the protein level of SelS, while three levels of dietary Se decreased (*p* < 0.05) the protein abundance of SelF. DOS and Se exhibited limited (*p* > 0.05) impact on the protein expression of DIO2.

## 4. Discussion

OS is a key factor of many diseases in mammals, especially those related to metabolism. As the primary metabolic organ, the liver is more susceptible to OS. The current leading theories posit that OS suppresses antioxidant capacity and causes liver injury, thus affecting liver lipid metabolism [4,34]. In this study, a long-term OS was administered to the growing-finishing pigs using oxidized diet. Although DOS exhibited no impacts on the liver weight and liver index (Figure 1), it tended to reduce the liver antioxidant capacity (Figure 1). Selenium is considered to be an important antioxidant, Se concentration in tissues is closely regulated by dietary Se levels, and the bioavailability of organic Se source is higher than the inorganic Se [21,35]. The results in this study revealed that OH-SeMet supplementation increased (*p* < 0.05) liver Se concentration in a dose dependent pattern (Figure 1B), and the pigs that received Se supplementation showed a higher liver weight and liver index (Figure 1A). Besides, dietary Se supplementation improved liver antioxidant capacity, which is mainly reflected by the elevated activity of GSH-Px, T-AOC and T-SOD (Figure 1). Based on these results, it is logical to expect that OH-SeMet plays a unique role in improving liver health.

Further study revealed that DOS increases serum ALT and ALP levels and decreases se rum TG concentration (Table 1). It is well known that ALT and ALP are the key biomarkers that reflect liver injury, and their elevated levels are usually accompanied by changes in liver metabolism [36,37]. Serum TG level is closely related to lipid metabolism, and high serum TG concentration is usually accompanied by fatty liver [38]. Our results suggest that DOS mediates liver damage and causes lipid metabolism disorders, which is consistent with previous studies [4,5,6]. Previous studies found that additional Se supplementation has limited effects on lipid metabolism, except for Se deficiency or high levels of Se (Se > 1 mg/kg) [22,23,24]. Interestingly, in this study additional Se supplementation decreased the ALT and ALP levels and restored the TG concentration in the serum. Therefore, it is reasonable to expect that Se may improve lipid metabolism by alleviating liver damage.

To verify whether the dietary OH-SeMet supplementation has a relieving effect on liver lipid metabolism under DOS, we further determined the activity of lipid metabolism related enzyme in the liver of pigs (Figure 2A–D). Although DOS exhibited limited impact on the activity of ACC and FAS, it increased the activity of HL and tended to increase the activity of ATGL. HL and ATGL are key enzymes that mediate lipolysis. HL is synthesized in liver parenchymal cells and released into plasma, mainly acting on TG to prevent its accumulation [39]. While ATGL specifically hydrolyzes the first ester bond of TG and is considered to be the rate-limiting enzyme in the TG hydrolysis [40]. The increased levels of HL and ATGL are consistent with the decreased TG concentration in the serum (Table 1), which confirms DOS causes lipid metabolic disorders. Se supplementation affected the activity of liver lipid metabolism related enzyme, such as increases in FAS activity and recovering of HL and ATGL activity (Figure 2). FAS has gained attention as an important regulator of the process of fatty acid chain extension [41]. These results support that the supplementation of OH-SeMet can relieve liver lipid metabolism disorders under DOS.

Previous studies indicate that lipid metabolism genes were affected or involved in redox regulation [42,43,44]. To comprehensively analyze the effects of DOS and Se on liver lipid metabolism, we further determined the expression of lipid metabolism related genes (Figure 2E). Interestingly, DOS generally down-regulated the expression of lipid synthesis-related genes except *DGAT1* and *G6PD*, while it up-regulated the expression of lipidolysis-related genes; these results are consistent with the previous findings [6,45]. Above those lipid synthesis-related genes, SCD is an ER protein that catalyzes the synthesis of unsaturated fatty acids [46], DGAT2 binds to the ER and mediates the synthesis of TG [47], FASN regulates the process of fatty acid chain extension [41], and those proteins play a crucial role in the process of fatty acid synthesis and carbon chain extension. As a key nuclear transcription factor, SREBP1c was mainly expressed in mammalian liver and adipose tissues that induces lipid synthesis [48]. For those lipidolysis-related genes, ACADL catalyzes the initial steps of β-oxidation of long-chain fatty acids [49], HSL and ATGL catalyze the hydrolysis of TG [50]. CPT1 contains two subtypes, CPT1a and CPT1b, both located at the inner mitochondrial membrane, and could transfer fatty acids to the mitochondria for β-oxidation [51]. ECH1, ACAA1/2 and ACOX1 are all located in the mitochondria, and they play a synergistic role in the β-oxidation process, ultimately catalyzing the decomposition of acetyl-CoA to produce ATP, carbon dioxide and water [52,53]. The downregulation of lipid synthesis-related genes and up-regulation of lipidolysis-related genes revealed that DOS-induced lipid metabolism disorders mainly manifested as inhibition of lipid synthesis and promotion of lipid decomposition. Dietary Se supplementation affected the expression of lipid metabolism genome, which is mainly reflected in facilitating the expression of lipid synthesis-related genes and suppressing the mRNA abundance of lipidolysis-related genes (Figure 2E). These results ulteriorly revealed that Se contributes to liver lipid metabolism regulation under DOS.

As a membrane-encapsulated organelle, the ER plays a unique role in lipid synthesis and transportation [7,8]; ER homeostasis is essential for lipid metabolism. However, the ER is highly sensitive to intracellular or extracellular stimuli, and OS is usually accompanied by ER stress [2]. Under ER stress, abundant of unfolded protein accumulate in the ER and induce UPR activation; thus, UPR is the most representative biomarker of the ER stress [11]. The UPR is mediated by three ER transmembrane sensors: IRE1, PERK and ATF6 [13]. These three pathways control multiple downstream factors such as eIF2α, ATF4, CHOP, XBP1 and GRP78, thus contributing to the regulation of ER homeostasis. Stress generally increases the expression of these biomarkers [54]. Consistent with the previous studies, we found DOS up-regulated the mRNA expression of *eIF2α*, *ATF4*, *CHOP* and *ATF6* in the liver, and DOS also increased the abundance of eIF2α and XBP1 at the protein level (Figure 3), suggesting ER stress was induced by DOS. Surprisingly, the pigs that received Se supplementation showed lower levels of *PERK*, *e-IF2α*, *ATF4*, *CHOP*, *XBP1*, *ATF6* and *GRP78* mRNA, and lower eIF2α and XBP1 protein levels, but higher P-eIF2α protein level (Figure 3). XBP-1 and eIF2α exhibited similar effects, and XBP1 could enter the nucleus to activate UPR after being activated as XBP-1s, while eIF2α can be phosphorylated into P-eIF2α and weakens the level of cell translation [13]. Our results suggested that Se mitigates the DOS-induced ER stress.

Se exerts most of its known biological functions through selenocysteine-containing selenoproteins [25]. In the past few years, the biological functions of most selenoproteins have been illustrated, from anti-inflammatory and antioxidant effects to the production of thyroid hormones [55,56]. Our current studies on the selenium genome found that Se exerts its biological functions through the coordination of multiple selenoproteins rather than a single selenoprotein [33,57,58]. Hence, we further explored the effect of OH-SeMet on the mRNA expression of selenotranscriptome in the liver. The results showed that though DOS alone exhibits limited effect on selenotranscriptome, dietary Se supplementation especially at 0.3 and 0.9 mg/kg effectively increased the mRNA abundance of 22 selenoprotein-encoding genes (Figure 4A and Supplementary Figure S2). Most of the above-mentioned selenoproteins are involved in regulating redox homeostasis, such as the glutathione peroxidases family (GPXs) and thioredoxin reductases family (TXNRDs) [59,60]. As an independent antioxidant system, GPXs received widespread attention with its ability to remove excessive ROS [61]. TXNRDs are the main substance of cells against disulfide damage. Besides, in view of the published studies, we found that several selenoproteins (DIOs, SelF, SelK, SelM, SelN, SelS and SelT) are located in the ER and contribute to ER calcium balance maintenance, processing of protein folding and antioxidant capacity improvement [62,63,64]. Therefore, the up-regulation of these selenoprotein-encoding genes indicated that selenoproteins may be involved into the antagonistic process of Se against OS and ER stress. To identify the major selenoproteins that resist ER stress and regulate lipid metabolism, principal component analysis was performed on the selenotranscriptome, 11 selenoprotein genes (*GPX1*, *GPX4*, *SELENOH*, *SELENOP*, *SELENOW*, *DIO2*, *SELENOF*, *SELENOK*, *SELENOM*, *SELENOS* and *SELENOT*) were selected, and 6 (*DIO2*, *SELENOF*, *SELENOK*, *SELENOM*, *SELENOS* and *SELENOT*) of them are ER resident proteins. Above these selenoproteins, GPX1 and GPX4 could directly remove the excessive ROS and improve the antioxidant capacity [59,65]. SelH and SelW are proposed to possess a thioredoxin-like fold structure, suggesting a redox function [66]. SelP transports Se from plasma to target organs, thus affecting the expression of all selenoproteins [67]. The up-regulation of those selenoprotein-encoding genes may imply a physiological necessity for the constant production of selenoproteins in liver to cope with DOS.

ER resident selenoproteins are essential for ER homeostasis. SelF participates in the protein quality control by mediating disulfide bond formation [68], and the protein level of SelF is generally up-regulated by stress conditions [69]. SelK is localized to ER membrane, and though the specific function of SelK is not yet clear, SelK silencing leads to ER stress and autophagy [62]. As a central component of retro-translocation channel in endoplasmic reticulum-associated protein degradation (ERAD), SelS contribute to the protein folding process [70]. Emerging evidence shows that SelT suppresses OS and apoptosis, and SelT silencing induces high ROS level in kidney cells [71]. SelM is a selenocysteine containing protein with redox activity, which improves antioxidant capacity and relieves ER stress [72]. Deiodinase (DIOs) is mainly involved in thyroid hormone activity regulation, while the roles DIO2 in ER homeostasis are still unknown. The up-regulation of these selenoprotein-encoding genes suggested that OH-SeMet alleviates ER stress and maintains ER homeostasis through ER resident selenoproteins. To verify the results of these selenoprotein-encoding genes, some selenoproteins were selected for western blot analysis. DOS suppressed the protein abundance of GPX1 and GPX4, increasing the protein level of SelF (Figure 5). Dietary Se supplementation showed a mitigating effect, which is mainly reflected in increasing the GPX1, GPX4 and SelS levels, and decreasing the SelF level. These selenoproteins’ abundance is basically consistent with mRNA expression, except for SelF. The possible reason is that stress increased the protein levels of SelF to relieve stress [69], and additional Se promotes the relief of stress and recovers the SelF levels. Interestingly, inconsistent with mRNA levels, the protein levels of DIO2 were not affected by DOS and dietary Se, suggesting that DIO2 has a limited effect on the process of ER homeostasis regulation. Based on the above results, it is very well established that OH-SeMet regulates lipid metabolism by alleviating OS and ER stress through selenoproteins.

Selenium deficiency or excess cause lipid metabolism disorder [22,23,24], indicating that selenoproteins potentially regulate lipid metabolism. Our interest herein has been to explore the correlations between lipid metabolism-related genes and changes of 11 major selenoprotein-encoding genes’ expression under DOS (Figure 4D). We found that the increased expressions of 11 selenoprotein-encoding genes were positively correlated with the expression of lipid synthesis-related genes under DOS, which is mainly reflected in increasing *FASN*, *SCD* and *SREBP1c* levels. On the other hand, the increased expressions of 11 selenoprotein-encoding genes were negatively correlated with the expression of lipid decomposition-related genes, which is mainly reflected in decreasing in *ACAA1*, *ACAA2*, *ACOX1*, *CPT1a* and *ECH1* levels. Our results are consistent with the previous study that porcine obesity is mediated by the regulation of selenoprotein-encoding genes via the expression of lipid metabolism-related genes or vice versa [32]. Based on these results, it is logical to expect that selenoproteins regulate lipid metabolism homeostasis by affecting the expression of lipid metabolism-related enzymes under DOS.

## 5. Conclusions

**Figure 6:**
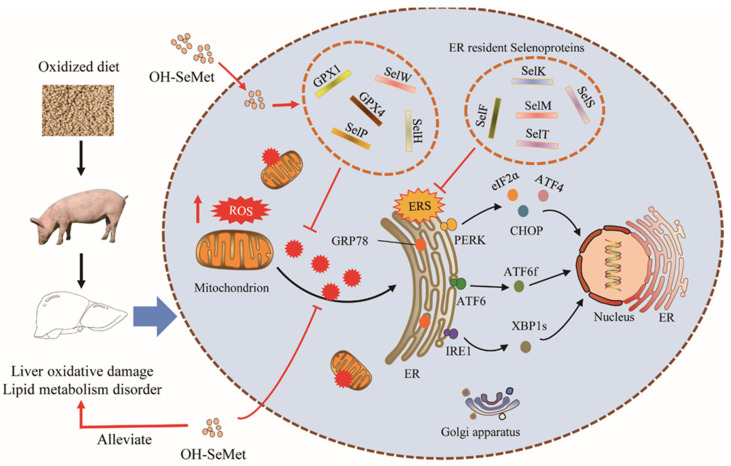
The proposed mechanism of OH-SeMet alleviates hepatic lipid metabolism disorder of pigs induced by oxidative stress.

The results of our study suggest the DOS induced liver damage leads to lipid metabolism disorder through activation of ER stress and UPR, and supplementation with Se in form of OH-SeMet beyond nutritional requirement support liver antioxidant capacity and mitigate ER stress by regulating the expression of selenotranscriptome, thus alleviating liver lipid metabolism disorders. Some key selenoproteins such as GPXs and ER resident selenoproteins play a crucial role in this process (Figure 6). Correlation analysis reveals that selenoproteins regulate lipid metabolism homeostasis by affecting the expression of few key lipid metabolism-related enzymes. This basic research contributes to better understanding on the potential protective mechanism of OH-SeMet in resisting DOS induced hepatic lipid metabolism disorder, which may contribute to identifying a possible therapeutic targeting/application in the field of OS dependent hepatic lipid metabolism disorder.

## Figures and Tables

**Table 1:** Effects of dietary oxidative stress and OH-SeMet supplementation on serum biochemical indicators of growing-finishing pigs.

Item	CON	DOS	DOS + 0.3 Se	DOS + 0.6 Se	DOS + 0.9 Se	ANOVA *p* Value
ALT/(U/L)	21.32 ± 3.53 ^ab^	24.00 ± 1.65 ^b^	20.92 ± 3.37 ^ab^	19.02 ± 3.29 ^ab^	17.11 ± 2.33 ^a^	0.008
AST/(U/L)	11.00 ± 0.59	12.97 ± 1.78	9.10 ± 2.56	10.89 ± 2.76	10.13 ± 2.19	0.098
ALP/(mmol/L)	56.67 ± 7.56 ^ab^	64.20 ± 7.78 ^b^	46.83 ± 6.41 ^a^	56.60 ± 6.47 ^ab^	54.20 ± 7.11 ^ab^	0.025
TP/(g/L)	41.08 ± 4.87	40.18 ± 4.60	40.60 ± 6.23	45.88 ± 4.27	43.03 ± 5.79	0.247
ALB/(g/L)	27.09 ± 3.24	26.40 ± 3.53	25.55 ± 2.60	29.40 ± 3.21	27.31 ± 3.03	0.238
GLB/(g/L)	13.99 ± 1.80	13.78 ± 1.49	15.05 ± 4.28	16.48 ± 3.56	15.71 ± 3.50	0.460
TG/(mmol/L)	0.51 ± 0.07 ^ab^	0.41 ± 0.05 ^a^	0.52 ± 0.08 ^ab^	0.60 ± 0.05 ^b^	0.56 ± 0.09 ^b^	0.001
TC/(mmol/L)	1.61 ± 0.22	1.73 ± 0.15	1.71 ± 0.19	1.68 ± 0.20	1.70 ± 0.15	0.838
HDL-C/(mmol/L)	0.81 ± 0.12	0.77 ± 0.06	0.81 ± 0.12	0.81 ± 0.08	0.82 ± 0.06	0.938
LDL-C/(mmol/L)	0.94 ± 0.15	1.05 ± 0.15	0.92 ± 0.11	1.00 ± 0.15	1.04 ± 0.15	0.457

ALT, alanine aminotransferase; AST, aspartate aminotransferase; ALP, alkaline phosphatase; TP, total protein; ALB, albumin; GLB, globulin; TG, triacylglycerol; TC, total cholesterol; LDL-C, low density lipoprotein cholesterin; HDL-C, high density lipoprotein cholesterin. Results were shown as mean ± SD (*n* = 6), different letters indicate ANOVA *p* value less than 0.05 which represent there is a significant difference between groups.

## Data Availability

Data is contained within the article and supplementary material.

## Supplementary Materials

The following supporting information can be downloaded at: https://www.mdpi.com/article/10.3390/antiox11030552/s1, Figure S1: Diet selenium concentration; Figure S2: The expression of selenotranscriptome in the liver; Table S1: Composition and nutrient levels of the basal diet; Table S2: The oxidation Characteristics of the diets; Table S3: Primers used for the Q-PCR; Table S4: The correlation analysis between the major selenoprotein-encoding genes and lipid metabolism markers.
