# Supplementary material for: Hydroxy Selenomethionine Alleviates Hepatic Lipid Metabolism Disorder of Pigs Induced by Dietary Oxidative Stress via Relieving the Endoplasmic Reticulum Stress
Source: Antioxidants (Basel). 2022 Mar 15;11(3):552. doi: 10.3390/antiox11030552 (PMC8945048; doi:10.3390/antiox11030552)
Supplement: Supplementary file 1 [file antioxidants-11-00552-s001.zip › antioxidants-1622562-Supplementary Tables.pdf]

Supplementary Information

Table S1. Composition and nutrient levels of the basal diet (air-dry basis, %)

Ingredients	Phase	
	25-50 kg	> 50 kg
Corn	73.610	70.525
Soybean meal	17.900	6.500
Fish meal	2.700	-
Rapeseed dregs	-	6.500
DDGS	-	8.000
Wheat bran	-	1.000
Corn oil	2.000	3.000
Fish oil	1.000	2.000
$CaHPO_4$	0.670	0.550
Limestone	0.930	0.900
NaCl	0.300	0.200
L-lysine HCl	0.440	0.470
DL-Methionine	0.100	0.030
L-Threonine	0.120	0.090
L-Tryptophan	0.030	0.035
Premix[1]	0.200	
Premix[2]		0.200
Total	100	100
Nutrient composition		
DE (Mcal/kg)	3.40	3.48
CP (%)	15.69	13.64
Ca (%)	0.66	0.53
AP (%)	0.31	0.24
SID Lys (%)	0.98	0.74
SID Met+cys (%)	0.54	0.43
SID Thr (%)	0.59	0.47
SID Trp (%)	0.17	0.14

Note: (1) Premix[1] for 25-50 kg pig provided (per kg): Cu ($CuSO_4 \cdot 5H_2O$), 4 m; I (KI), 0.14 mg; Fe ($FeSO_4 \cdot 7H_2O$), 60 mg; Mn ($MnSO_4 \cdot H_2O$), 2 mg; Zn ($ZnSO_4 \cdot H_2O$), 60 mg; 50% Choline chloride, 75 mg; Vitamin A, 1500 IU; Vitamin D3, 500 IU; Vitamin E, 4 mg; Vitamin K3, 0.5 mg; Vitamin B1, 0.5 mg; Vitamin B2, 1.25 mg; Vitamin B6, 0.6 mg; Vitamin B12, 6 μg; D-biotin, 25 μg; D-pantothenic acid, 2.5 mg; Folic acid, 0.25 mg; Nicotinamide, 5 mg.

(2) Premix[2] for > 50 kg pig provided (per kg): Cu ($CuSO_4 \cdot 5H_2O$), 7 mg; I (KI), 0.2 mg; Fe ($FeSO_4 \cdot 7H_2O$), 100 mg; Mn ($MnSO_4 \cdot H_2O$), 4 mg; Zn ($ZnSO_4 \cdot H_2O$), 50 mg; 50% Choline chloride, 200 mg; Vitamin A, 450 IU; Vitamin D3, 150 IU; Vitamin E, 1.2 mg; Vitamin K3, 0.15 mg; Vitamin B1, 0.15 mg; Vitamin B2, 0.375 mg; Vitamin B6, 0.18 mg; Vitamin B12, 1.8 μg; D-biotin, 7.5 μg; D-pantothenic acid, 0.75 mg; Folic acid, 75 μg; Nicotinamide, 1.5 mg.

Table S2 The oxidation Characteristics of the diets

Item	CON	DOS	DOS + 0.3 Se	DOS + 0.6 Se	DOS + 0.9 Se
Diet for 25-50 kg					
AV (mg KOH/100 g)	56.70	238.90	238.40	233.30	240.20
POV (mmol O_2/kg)	19.60	433.60	415.70	429.20	414.60
IV (g I/100 g)	126.70	81.20	83.90	83.50	82.10
SV (mg KOH/g)	182.10	237.70	240.60	248.30	242.70
Diet for > 50 kg					
AV (mg KOH/100 g)	68.00	252.60	253.70	264.60	257.40
POV (mmol O_2/kg)	31.80	519.20	523.60	537.80	534.20
IV (g I/100 g)	111.70	73.40	73.60	74.70	72.80
SV (mg KOH/g)	195.40	261.30	265.70	268.20	266.70

AV, acid value; POV, peroxide value; IV, iodine value; SV, saponification value.

Table S3. Primers used for the Q-PCR

Gene	Accession number	Primer pairs (5′ to 3′ direction)
**Housekeeping control genes**		
β-Actin	AY550069	F:CCCAAAGCCAACCGTGAGAA<br>R:CCACGTACATGGCTGGGGTG
Gapdh	NM_001206359	F:CAGCAATGCCTCCTGTACCA<br>R:CCACGATGCCGAAGTTGTC
**Lipid metabolic-related genes**		
ACACA	NM_001114269.1	F:CAAGACCACCAACGCGAAA<br>R:GGCAAATGGGAGGCAATAAGA
FASN	NM_001099930	F:CAAGCAGGCGAACACGATG<br>R:AAGGGAAGCAGGGTTGATGC
SCD	NM_213781.1	F:CAAACACCCAGCCGTCAAAG<br>R:AGCTTCACCCCAGCAATACC
DGAT1	NM_214051.1	F:ACAAGGACAAGGACGGACAC<br>R:ATGCCACGGTAGTTGCTGAA
DGAT2	NM_001160080.1	F:ATGCCCTACTCCAAGCCCA<br>R:CCGAACTTGGTCTTGTGCTG
G6PD	XM_003360515.5	F:TGGTGGCTGTTCCGAGATG<br>R:GGCGTAGCCCACGATGTATG
SREBP1c	NM_214157.1	F:AAGCGGACGGCTCACAATG<br>R:GCAAGACGGCGGATTTATTCA
CPT1a	NM_001129805.1	F:TGGTGTCCAAATACCTCGCC<br>R:CCTCCGCTCGACACATACTC
CPT1b	NM_001007191.1	F:GCGCCGCGTGAAAGC<br>R:GATCCCAGTCCCGGTCATG
CPT2	NM_001246243.1	F:CGAGTGTTCCGAGTACCACAA<br>R:CAAGTGTCGGTCAAAGCCCT
HSL	NM_214315	F:CTCGTGGCTCAACTCCTTCCT

		R:GCCGCATTGGCTCTTCTGT
LIPC	NM_001143714.1	F:AGCAGGAGAAACCCAGCAAA<br>R:CCTGGGTTGGGTGAAGTTGT
ATGL1	NM_001098605.1	F:CAAGAGCACCATCACCGTGTCC<br>R:AGGCCGTCCCTGTAACCCT
ACAA1	XM_021071664.1	F:TCTGCGTGGGAAATGTGCTT<br>R:CTCAGAAACTGGGCGATTCG
ACAA2	NM_001167638.1	F:TCGTGGGCTATTTTGCGTCT<br>R:TCCTGCTTTCTTCAGTGCCC
ACOX1	NM_001101028.1	F:GCCGCCGAGAGATCGAAA<br>R:GGATGTCCTCGCTGCACAAA
ACADL	NM_213897.1	F:GCGTGGCTTATGACTGTGTG<br>R:ACTCGGGCATCCACATAAGC
ECH1	NM_001044607.2	F:GCTAAACCGGCCAGAGAAGA<br>R:AGATGTCCGAAGCCATGTCC
**Endoplasmic reticulum stress biomarkers**		
PERK	XM_003124925.4	F:CTGTACCCATTCGGCACTCA<br>R:ATGTCCTGCACCATCGCAAA
eIF2α	XM_001928339.4	F:AATAGGCGTTTGACCCCACA<br>R:TGTTCTCTCCAGGGTGGTTG
ATF4	NM_001123078.1	F:ATGCCCTGTCGGGTATAGATGA<br>R:ATCCAACGTGGCCAAAAGC
CHOP	NM_001144845.1	F:GGAAATGAGGAGGAGTCAAAAACC<br>R:CTCAGTCAGCCAAGCCAGAGA
IRE1	XM_005668695.3	F:CTGAGCGAAGACTGCAAGGA<br>R:GAGTATGTTGGCCTGACGCT
XBP-1	NM_001142836.1	F:CAGACTGCCAGAGACCGAAAGA<br>R:TCTTCCAAATCTACCACTTGTTGCT
ATF6	XM_021089516.1	F:GGGAGTGAGCTGCAGGTGTATT<br>R:TCTGCGGATGGCTTCAAAGA
GRP78	XM_001927795.7	F:TGGGAAAGAAGGTTACTCATGCA<br>R:CTGGCGTTGGGCATCATT
**Selenoprotein-encoding genes**		
DIO1	NM_001001627	F:CATGGCCAAGAACCCTCACT<br>R:CCAGAAATACTGGGCACTGAAGA
DIO2	NM_001001626	F:CGCTGCATCTGGAAGAGCTT<br>R:TGGAATTGGGTGCATCTTCA
DIO3	NM_001001625	F:TGAAGTGGAGCTCAACAGTGATG<br>R:TGTCGTCAGACACGCAGATAGG
GPX1	NM_214201	F:GATGCCACTGCCCTCATGA<br>R:TCGAAGTTCCATGCGATGTC
GPX2	NM_001115136	F:AGAATGTGGCCTCGCTCTGA<br>R:GGCATTGCAGCTCGTTGAG
GPX3	NM_001115155	F:CCGGTTCCTGTTTTCCAAATT<br>R:TGCACTGCAGGAAGAGTTTGAA
GPX4	NM_214407	F:TGAGGCAAGACGGAGGTAAACT<br>R:TCCGTAAACCACACTCAGCATATC
GPX6	NM_001137607	F:GAGCTGAAGCCTTTTGGTGTAGTT

		R:CTTTGCTGGTTCTTGTTTTCCA
MSRB1	NM_001097460	F:ATCCCTAAAGGCCAAGAATCATC<br>R:GGCCACCAAGCAGTGTTCA
SELENOF	NM_001085443	F:ACAGCCCTGCCAAGCAGAT<br>R:AACAGGGAGGCTGGGTAACAC
SELENOH	NM_001184948	F:TGGTGGAGGAGCTGAAGAAGTAC<br>R:CGTCATAAATGCTCCAACATCAC
SELENOI	NM_001244662	F:GATGGTGTGGATGGAAAGCAA<br>R:GCCATGGTCAAAGAGTTCTCCTA
SELENOK	NM_001044553	F:CAGGAAACCCCCCTAGAAGAA<br>R:CTCATCCACCGGCCATTG
SELENOM	NM_001161648	F:CAGCTGAATCGCCTCAAAGAG<br>R:GAGATGTTTCATGACCAGGTTGTG
SELENON	XM_021095600	F:ACCTGGTCCCTGGTGAAAGAG<br>R:AGGCCAGCCAGCTTCTTGT
SELENOO	NM_001201431	F:CTTCCGACCCCAGATGGAT<br>R:GGTTCGACTGTGCCAGCAT
SELENOP	NM_001134823	F:AACCAGAAGCGCCAGACACT<br>R:TGCTGGCATATCTCAGTTCTCAGA
SELENOS	NM_001164113	F:GAGGCAGAGGCACCTGGAT<br>R:CTGCTAAAGCCTCCTGTCGTTT
SELENOT	NM_001163408	F:GGCTTAATAATCGTTGGCAAAGA<br>R:TGGCCCCATTGCCAGATA
SELENOV	NM_001166045	F:CACTGGTCGCCAATGGATTC<br>R:AGTGGCCAACGGAGAAAGC
SELENOW	NM_213977	F:CACCCCTGTCTCCCTGCAT<br>R:GAGCAGGATCACCCCAAACA
SEPHS2	NM_001093735	F:TGGCTTGATGCACACGTTTAA<br>R:TGCGAGTGTCCCAGAATGC
TXNRD1	NM_214154	F:GATTTAACAAGCGGGTCATGGT<br>R:CAACCTACATTCACACACGTTCCT
TXNRD2	NM_001168702	F:TCTTGAAAGGCGGAAAAGAGAT<br>R:TCGGTCGCCCTCCAGTAG
TXNRD3	XM_013982024	F:GTGCCCTACGTTTATGCTGTTG<br>R:TCCGAGCCACCAGCTTTG

Table S4. The correlation analysis between the major selenoprotein-encoding genes and lipid metabolism markers

Correlations

		GPX1	GPX4	SELENOH	SELENOP	SELENOW	DIO2	SELENOF	SELENOK	SELENOM	SELENOS	SELENOT	FASN	SCD	DGAT1	DGAT2	G6PD	SREBP1	ACAA1	ACAA2	ACADL	ACOX1	ATGL1	CPT1a	CPT1b	ECH1	HSL
GPX1	Pearson Correlation	1	.785**	.676**	.644**	.869**	.628**	.565**	.860**	.696**	.568**	.836**	.379*	.711**	0.089	0.075	0.113	.699**	-.595**	-.526**	-.416*	-.588**	-.404*	-.602**	-0.298	-.597**	-.424*
	Sig. (2-tailed)		0.000	0.000	0.000	0.000	0.000	0.001	0.000	0.000	0.001	0.000	0.039	0.000	0.640	0.694	0.554	0.000	0.001	0.003	0.022	0.001	0.027	0.000	0.110	0.000	0.020
	N	30	30	30	30	30	30	30	30	30	30	30	30	30	30	30	30	30	30	30	30	30	30	30	30	30	30
GPX4	Pearson Correlation	.785**	1	.598**	.626**	.834**	.558**	.605**	.859**	.762**	.695**	.891**	0.287	.580**	0.230	0.030	0.042	.585**	-.518**	-.478**	-0.303	-.556**	-0.300	-.540**	-0.160	-.544**	-.377*
	Sig. (2-tailed)	0.000		0.000	0.000	0.000	0.001	0.000	0.000	0.000	0.000	0.000	0.124	0.001	0.221	0.873	0.826	0.001	0.003	0.007	0.103	0.001	0.107	0.002	0.398	0.002	0.040
	N	30	30	30	30	30	30	30	30	30	30	30	30	30	30	30	30	30	30	30	30	30	30	30	30	30	30
SELENOH	Pearson Correlation	.676**	.598**	1	.443*	.874**	.680**	.606**	.733**	.714**	.550**	.630**	.422*	.623**	-0.024	0.005	0.212	.660**	-.522**	-.580**	-.448*	-.637**	-.424*	-.651**	-0.291	-.620**	-0.284
	Sig. (2-tailed)	0.000	0.000		0.014	0.000	0.000	0.000	0.000	0.000	0.002	0.000	0.020	0.000	0.898	0.979	0.261	0.000	0.003	0.001	0.013	0.000	0.020	0.000	0.118	0.000	0.128
	N	30	30	30	30	30	30	30	30	30	30	30	30	30	30	30	30	30	30	30	30	30	30	30	30	30	30
SELENOP	Pearson Correlation	.644**	.626**	.443*	1	.675**	.710**	0.242	.691**	.467**	.695**	.769**	0.290	.650**	-0.005	0.020	-0.124	.577**	-.370*	-.465**	-0.190	-.496**	-.441*	-.508**	-0.271	-.543**	-.493**
	Sig. (2-tailed)	0.000	0.000	0.014		0.000	0.000	0.198	0.000	0.009	0.000	0.000	0.120	0.000	0.981	0.915	0.515	0.001	0.044	0.010	0.313	0.005	0.015	0.004	0.147	0.002	0.006
	N	30	30	30	30	30	30	30	30	30	30	30	30	30	30	30	30	30	30	30	30	30	30	30	30	30	30
SELENOW	Pearson Correlation	.869**	.834**	.874**	.675**	1	.782**	.649**	.889**	.838**	.658**	.881**	.459*	.785**	0.058	0.007	0.177	.780**	-.628**	-.635**	-.491**	-.737**	-.452*	-.757**	-0.319	-.743**	-.406*
	Sig. (2-tailed)	0.000	0.000	0.000	0.000		0.000	0.000	0.000	0.000	0.000	0.000	0.011	0.000	0.763	0.970	0.349	0.000	0.000	0.000	0.006	0.000	0.012	0.000	0.086	0.000	0.026
	N	30	30	30	30	30	30	30	30	30	30	30	30	30	30	30	30	30	30	30	30	30	30	30	30	30	30
DIO2	Pearson Correlation	.628**	.558**	.680**	.710**	.782**	1	.457*	.700**	.608**	.417*	.705**	0.333	.790**	0.010	0.008	0.036	.761**	-.489**	-.583**	-0.359	-.695**	-.505**	-.729**	-.373*	-.732**	-.481**
	Sig. (2-tailed)	0.000	0.001	0.000	0.000	0.000		0.011	0.000	0.000	0.022	0.000	0.072	0.000	0.957	0.967	0.852	0.000	0.006	0.001	0.051	0.000	0.004	0.000	0.042	0.000	0.007
	N	30	30	30	30	30	30	30	30	30	30	30	30	30	30	30	30	30	30	30	30	30	30	30	30	30	30
SELENOF	Pearson Correlation	.565**	.605**	.606**	0.242	.649**	.457*	1	.443*	.830**	0.279	.610**	.367*	.670**	-0.026	0.159	0.289	.594**	-.581**	-.578**	-.555**	-.645**	-0.251	-.621**	-0.046	-.608**	-0.038
	Sig. (2-tailed)	0.001	0.000	0.000	0.198	0.000	0.011		0.014	0.000	0.135	0.000	0.046	0.000	0.893	0.401	0.122	0.001	0.001	0.001	0.001	0.000	0.181	0.000	0.810	0.000	0.842
	N	30	30	30	30	30	30	30	30	30	30	30	30	30	30	30	30	30	30	30	30	30	30	30	30	30	30
SELENOK	Pearson Correlation	.860**	.859**	.733**	.691**	.889**	.700**	.443*	1	.657**	.731**	.841**	0.323	.601**	0.208	-0.125	0.060	.707**	-.497**	-.490**	-0.278	-.565**	-.429*	-.584**	-0.304	-.576**	-.510**
	Sig. (2-tailed)	0.000	0.000	0.000	0.000	0.000	0.000	0.014		0.000	0.000	0.000	0.082	0.000	0.270	0.509	0.752	0.000	0.005	0.006	0.137	0.001	0.018	0.001	0.102	0.001	0.004
	N	30	30	30	30	30	30	30	30	30	30	30	30	30	30	30	30	30	30	30	30	30	30	30	30	30	30
SELENOM	Pearson Correlation	.696**	.762**	.714**	.467**	.838**	.608**	.830**	.657**	1	.429*	.727**	.454*	.781**	0.083	-0.013	0.292	.746**	-.614**	-.606**	-.558**	-.710**	-0.307	-.717**	-0.161	-.701**	-0.100
	Sig. (2-tailed)	0.000	0.000	0.000	0.009	0.000	0.000	0.000	0.000		0.018	0.000	0.012	0.000	0.663	0.948	0.117	0.000	0.000	0.000	0.001	0.000	0.099	0.000	0.394	0.000	0.599
	N	30	30	30	30	30	30	30	30	30	30	30	30	30	30	30	30	30	30	30	30	30	30	30	30	30	30
SELENOS	Pearson Correlation	.568**	.695**	.550**	.695**	.658**	.417*	0.279	.731**	.429*	1	.781**	0.188	.446*	0.081	-0.126	-0.237	.440*	-0.240	-0.299	-0.081	-0.270	-.411*	-0.294	-0.135	-0.296	-.457*
	Sig. (2-tailed)	0.001	0.000	0.002	0.000	0.000	0.022	0.135	0.000	0.018		0.000	0.321	0.014	0.670	0.507	0.208	0.015	0.202	0.109	0.669	0.149	0.024	0.115	0.476	0.112	0.011
	N	30	30	30	30	30	30	30	30	30	30	30	30	30	30	30	30	30	30	30	30	30	30	30	30	30	30
SELENOT	Pearson Correlation	.836**	.891**	.630**	.769**	.881**	.705**	.610**	.841**	.727**	.781**	1	0.279	.738**	0.059	0.040	-0.086	.679**	-.516**	-.533**	-0.358	-.576**	-.396*	-.598**	-0.244	-.608**	-.455*
	Sig. (2-tailed)	0.000	0.000	0.000	0.000	0.000	0.000	0.000	0.000	0.000	0.000		0.136	0.000	0.756	0.834	0.651	0.000	0.003	0.002	0.052	0.001	0.030	0.000	0.194	0.000	0.011
	N	30	30	30	30	30	30	30	30	30	30	30	30	30	30	30	30	30	30	30	30	30	30	30	30	30	30
FASN	Pearson Correlation	.379*	0.287	.422*	0.290	.459*	0.333	.367*	0.323	.454*	0.188	0.279	1	.587**	-0.104	0.082	0.258	.605**	-.709**	-.697**	-.728**	-.724**	-.619**	-.680**	-.392*	-.740**	-0.305
	Sig. (2-tailed)	0.039	0.124	0.020	0.120	0.011	0.072	0.046	0.082	0.012	0.321	0.136		0.001	0.585	0.667	0.169	0.000	0.000	0.000	0.000	0.000	0.000	0.000	0.032	0.000	0.101
	N	30	30	30	30	30	30	30	30	30	30	30	30	30	30	30	30	30	30	30	30	30	30	30	30	30	30
SCD	Pearson Correlation	.711**	.580**	.623**	.650**	.785**	.790**	.670**	.601**	.781**	.446*	.738**	.587**	1	-0.019	0.047	0.146	.835**	-.647**	-.699**	-.614**	-.793**	-.582**	-.806**	-0.331	-.821**	-0.318
	Sig. (2-tailed)	0.000	0.001	0.000	0.000	0.000	0.000	0.000	0.000	0.000	0.014	0.000	0.001		0.922	0.806	0.441	0.000	0.000	0.000	0.000	0.000	0.001	0.000	0.074	0.000	0.087
	N	30	30	30	30	30	30	30	30	30	30	30	30	30	30	30	30	30	30	30	30	30	30	30	30	30	30
DGAT1	Pearson Correlation	0.089	0.230	-0.024	-0.005	0.058	0.010	-0.026	0.208	0.083	0.081	0.059	-0.104	-0.019	1	-0.329	-0.011	0.046	0.204	.446*	.465**	0.149	0.326	0.168	0.189	0.160	0.117
	Sig. (2-tailed)	0.640	0.221	0.898	0.981	0.763	0.957	0.893	0.270	0.663	0.670	0.756	0.585	0.922		0.076	0.953	0.810	0.279	0.014	0.010	0.431	0.079	0.376	0.317	0.398	0.540
	N	30	30	30	30	30	30	30	30	30	30	30	30	30	30	30	30	30	30	30	30	30	30	30	30	30	30
DGAT2	Pearson Correlation	0.075	0.030	0.005	0.020	0.007	0.008	0.159	-0.125	-0.013	-0.126	0.040	0.082	0.047	-0.329	1	0.179	0.045	-.373*	-0.346	-0.211	-0.115	-0.025	-0.063	-0.143	-0.093	-0.230

	Sig. (2-tailed)	0.694	0.873	0.979	0.915	0.970	0.967	0.401	0.509	0.948	0.507	0.834	0.667	0.806	0.076		0.344	0.814	0.042	0.061	0.263	0.546	0.895	0.742	0.450	0.626	0.220
	N	30	30	30	30	30	30	30	30	30	30	30	30	30	30	30	30	30	30	30	30	30	30	30	30	30	30
G6PD	Pearson Correlation	0.113	0.042	0.212	-0.124	0.177	0.036	0.289	0.060	0.292	-0.237	-0.086	0.258	0.146	-0.011	0.179	1	0.142	-0.248	-0.175	-0.116	-0.250	0.069	-0.212	0.326	-0.188	0.183
	Sig. (2-tailed)	0.554	0.826	0.261	0.515	0.349	0.852	0.122	0.752	0.117	0.208	0.651	0.169	0.441	0.953	0.344		0.454	0.187	0.354	0.543	0.183	0.718	0.261	0.079	0.319	0.332
	N	30	30	30	30	30	30	30	30	30	30	30	30	30	30	30	30	30	30	30	30	30	30	30	30	30	30
SREBP1	Pearson Correlation	.699**	.585**	.660**	.577**	.780**	.761**	.594**	.707**	.746**	.440*	.679**	.605**	.835**	0.046	0.045	0.142	1	-.696**	-.678**	-.566**	-.750**	-.612**	-.754**	-.435*	-.774**	-.373*
	Sig. (2-tailed)	0.000	0.001	0.000	0.001	0.000	0.000	0.001	0.000	0.000	0.015	0.000	0.000	0.000	0.810	0.814	0.454		0.000	0.000	0.001	0.000	0.000	0.000	0.016	0.000	0.043
	N	30	30	30	30	30	30	30	30	30	30	30	30	30	30	30	30	30	30	30	30	30	30	30	30	30	30
ACAA1	Pearson Correlation	-.595**	-.518**	-.522**	-.370*	-.628**	-.489**	-.581**	-.497**	-.614**	-0.240	-.516**	-.709**	-.647**	0.204	-.373*	-0.248	-.696**	1	.857**	.742**	.776**	.487**	.765**	.485**	.834**	.549**
	Sig. (2-tailed)	0.001	0.003	0.003	0.044	0.000	0.006	0.001	0.005	0.000	0.202	0.003	0.000	0.000	0.279	0.042	0.187	0.000		0.000	0.000	0.000	0.006	0.000	0.007	0.000	0.002
	N	30	30	30	30	30	30	30	30	30	30	30	30	30	30	30	30	30	30	30	30	30	30	30	30	30	30
ACAA2	Pearson Correlation	-.526**	-.478**	-.580**	-.465**	-.635**	-.583**	-.578**	-.490**	-.606**	-0.299	-.533**	-.697**	-.699**	.446*	-0.346	-0.175	-.678**	.857**	1	.866**	.821**	.608**	.777**	.545**	.840**	.525**
	Sig. (2-tailed)	0.003	0.007	0.001	0.010	0.000	0.001	0.001	0.006	0.000	0.109	0.002	0.000	0.000	0.014	0.061	0.354	0.000	0.000		0.000	0.000	0.000	0.000	0.002	0.000	0.003
	N	30	30	30	30	30	30	30	30	30	30	30	30	30	30	30	30	30	30	30	30	30	30	30	30	30	30
ACADL	Pearson Correlation	-.416*	-0.303	-.448*	-0.190	-.491**	-0.359	-.555**	-0.278	-.558**	-0.081	-0.358	-.728**	-.614**	.465**	-0.211	-0.116	-.566**	.742**	.866**	1	.740**	.585**	.736**	.526**	.750**	0.263
	Sig. (2-tailed)	0.022	0.103	0.013	0.313	0.006	0.051	0.001	0.137	0.001	0.669	0.052	0.000	0.000	0.010	0.263	0.543	0.001	0.000	0.000		0.000	0.001	0.000	0.003	0.000	0.160
	N	30	30	30	30	30	30	30	30	30	30	30	30	30	30	30	30	30	30	30	30	30	30	30	30	30	30
ACOX1	Pearson Correlation	-.588**	-.556**	-.637**	-.496**	-.737**	-.695**	-.645**	-.565**	-.710**	-0.270	-.576**	-.724**	-.793**	0.149	-0.115	-0.250	-.750**	.776**	.821**	.740**	1	.626**	.925**	.384*	.934**	0.360
	Sig. (2-tailed)	0.001	0.001	0.000	0.005	0.000	0.000	0.000	0.001	0.000	0.149	0.001	0.000	0.000	0.431	0.546	0.183	0.000	0.000	0.000	0.000		0.000	0.000	0.036	0.000	0.051
	N	30	30	30	30	30	30	30	30	30	30	30	30	30	30	30	30	30	30	30	30	30	30	30	30	30	30
ATGL1	Pearson Correlation	-.404*	-0.300	-.424*	-.441*	-.452*	-.505**	-0.251	-.429*	-0.307	-.411*	-.396*	-.619**	-.582**	0.326	-0.025	0.069	-.612**	.487**	.608**	.585**	.626**	1	.615**	0.322	.616**	.440*
	Sig. (2-tailed)	0.027	0.107	0.020	0.015	0.012	0.004	0.181	0.018	0.099	0.024	0.030	0.000	0.001	0.079	0.895	0.718	0.000	0.006	0.000	0.001	0.000		0.000	0.083	0.000	0.015
	N	30	30	30	30	30	30	30	30	30	30	30	30	30	30	30	30	30	30	30	30	30	30	30	30	30	30
CPT1a	Pearson Correlation	-.602**	-.540**	-.651**	-.508**	-.757**	-.729**	-.621**	-.584**	-.717**	-0.294	-.598**	-.680**	-.806**	0.168	-0.063	-0.212	-.754**	.765**	.777**	.736**	.925**	.615**	1	.415*	.937**	.403*
	Sig. (2-tailed)	0.000	0.002	0.000	0.004	0.000	0.000	0.000	0.001	0.000	0.115	0.000	0.000	0.000	0.376	0.742	0.261	0.000	0.000	0.000	0.000	0.000	0.000		0.023	0.000	0.027
	N	30	30	30	30	30	30	30	30	30	30	30	30	30	30	30	30	30	30	30	30	30	30	30	30	30	30
CPT1b	Pearson Correlation	-0.298	-0.160	-0.291	-0.271	-0.319	-.373*	-0.046	-0.304	-0.161	-0.135	-0.244	-.392*	-0.331	0.189	-0.143	0.326	-.435*	.485**	.545**	.526**	.384*	0.322	.415*	1	.476**	.439*
	Sig. (2-tailed)	0.110	0.398	0.118	0.147	0.086	0.042	0.810	0.102	0.394	0.476	0.194	0.032	0.074	0.317	0.450	0.079	0.016	0.007	0.002	0.003	0.036	0.083	0.023		0.008	0.015
	N	30	30	30	30	30	30	30	30	30	30	30	30	30	30	30	30	30	30	30	30	30	30	30	30	30	30
ECH1	Pearson Correlation	-.597**	-.544**	-.620**	-.543**	-.743**	-.732**	-.608**	-.576**	-.701**	-0.296	-.608**	-.740**	-.821**	0.160	-0.093	-0.188	-.774**	.834**	.840**	.750**	.934**	.616**	.937**	.476**	1	.462*
	Sig. (2-tailed)	0.000	0.002	0.000	0.002	0.000	0.000	0.000	0.001	0.000	0.112	0.000	0.000	0.000	0.398	0.626	0.319	0.000	0.000	0.000	0.000	0.000	0.000	0.000	0.008		0.010
	N	30	30	30	30	30	30	30	30	30	30	30	30	30	30	30	30	30	30	30	30	30	30	30	30	30	30
HSL	Pearson Correlation	-.424*	-.377*	-0.284	-.493**	-.406*	-.481**	-0.038	-.510**	-0.100	-.457*	-.455*	-0.305	-0.318	0.117	-0.230	0.183	-.373*	.549**	.525**	0.263	0.360	.440*	.403*	.439*	.462*	1
	Sig. (2-tailed)	0.020	0.040	0.128	0.006	0.026	0.007	0.842	0.004	0.599	0.011	0.011	0.101	0.087	0.540	0.220	0.332	0.043	0.002	0.003	0.160	0.051	0.015	0.027	0.015	0.010	
	N	30	30	30	30	30	30	30	30	30	30	30	30	30	30	30	30	30	30	30	30	30	30	30	30	30	30

**. Correlation is significant at the 0.01 level (2-tailed).

*. Correlation is significant at the 0.05 level (2-tailed).
