# Supplementary figures and images for: Hydroxy Selenomethionine Alleviates Hepatic Lipid Metabolism Disorder of Pigs Induced by Dietary Oxidative Stress via Relieving the Endoplasmic Reticulum Stress
Source: Antioxidants (Basel). 2022 Mar 15;11(3):552. doi: 10.3390/antiox11030552 (PMC8945048; doi:10.3390/antiox11030552)

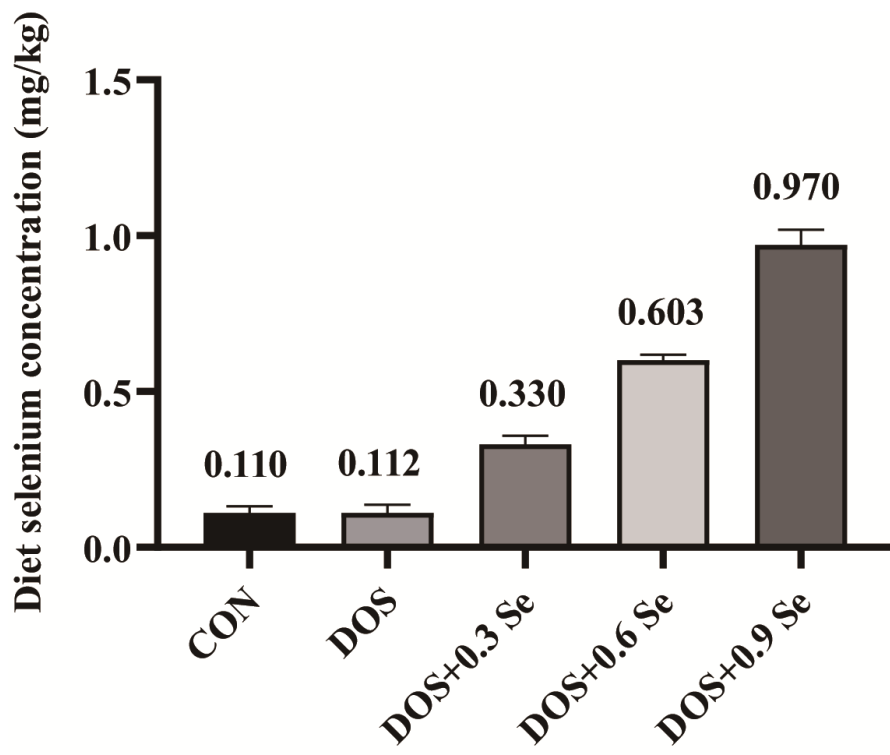

Supplementary Figure S1

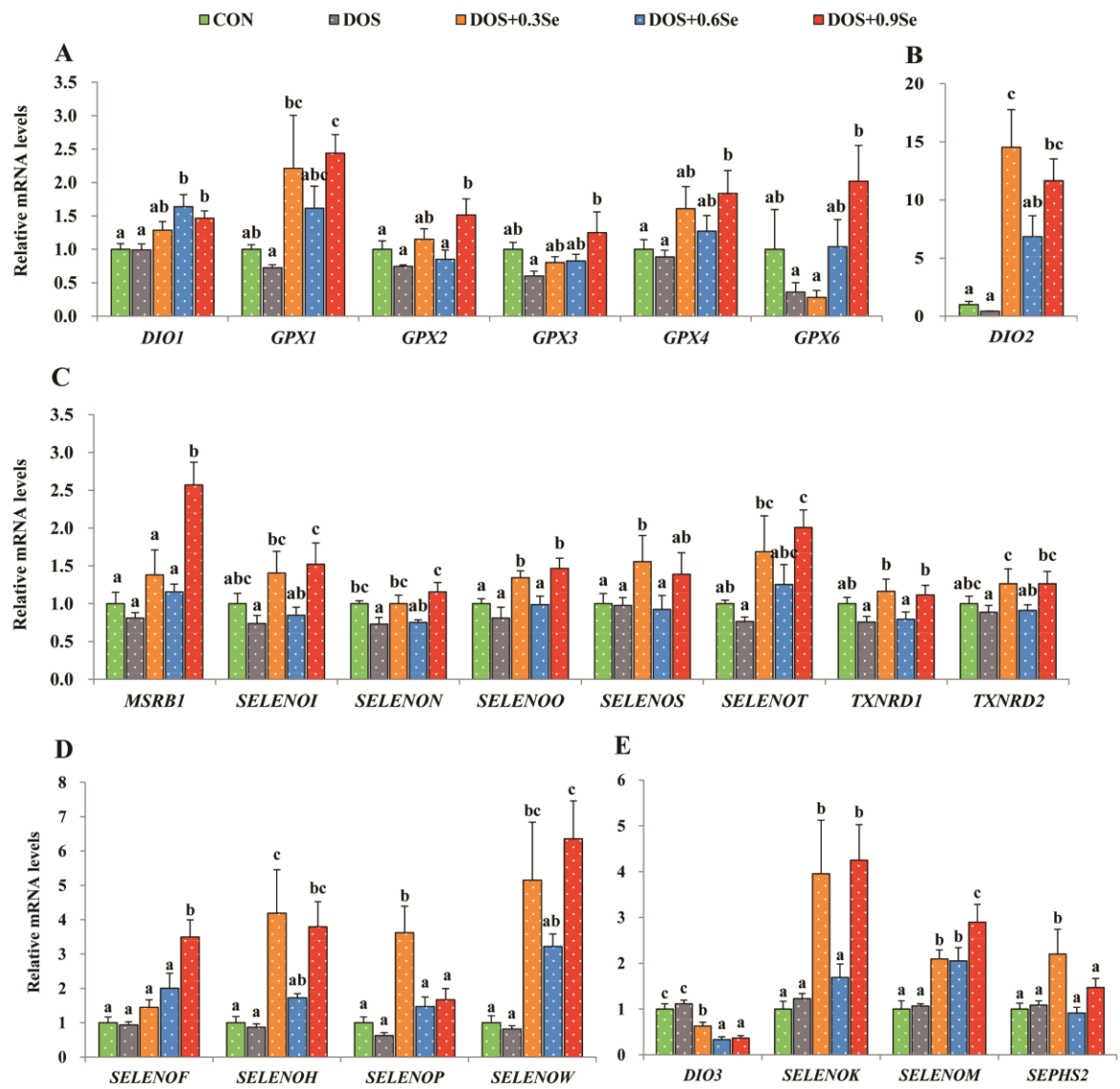

Supplementary Figure S2

Supplement: Supplementary file 1 [file antioxidants-11-00552-s001.zip › antioxidants-1622562-Supplementary Figures.pdf]
